# Work experiences of healthcare professionals in a shortage context: analysis of open-ended comments in a Swiss cohort (SCOHPICA)

**DOI:** 10.1186/s12913-025-12659-z

**Published:** 2025-04-09

**Authors:** Ingrid Gilles, Clara Le Saux, Emilie Zuercher, Jonathan Jubin, Léonard Roth, Annie Oulevey Bachmann, Isabelle Peytremann-Bridevaux

**Affiliations:** 1 Department of Epidemiology and Health Systems, Centre for Primary Care and Public Health (Unisante), University of Lausanne, 10 Route de la Corniche, Lausanne, 1010 Switzerland; 2 Human Resources Direction, Lausanne University Hospital, Lausanne, Switzerland; 3 La Source School of Nursing, HES-so University of Applied Sciences and Arts Western Switzerland, Lausanne, Switzerland

**Keywords:** Healthcare professionals, Workforce retention, Health policy, Education, Open-ended comments, Computer assisted analysis

## Abstract

**Background:**

Healthcare systems worldwide face critical shortages of healthcare professionals (HCPs), threatening care quality and system sustainability. In Switzerland, limited training capacities further worsen the situation. While factors, such as job satisfaction, work-life balance, and burnout, are well-documented, most studies focus on specific professions, limiting their generalizability. This study provides an interprofessional analysis of HCPs' experiences regarding their working conditions in the context of workforce shortages, identifying key challenges that could inform retention strategies.

**Methods:**

Open-ended comments from 1,811 participants in the HCPs part of the Swiss Cohort of Healthcare professionals and Informal caregivers (SCOHPICA-HCP), covering over 30 healthcare professions, were analyzed using computer-assisted textual analysis (IRaMuTeQ). Thematic classes were identified through hierarchical classification, and chi-square tests were conducted to examine associations with participant characteristics (e.g., profession, work setting, job satisfaction).

**Results:**

Three main themes emerged. First, participants highlighted gaps in training, including inadequate preparation for professional realities, limited career development opportunities, and challenges in diploma recognition. Second, systemic failures —such as staff shortages, inadequate wages, and administrative overload — were linked to stress, burnout, and declining care quality. Many participants perceived a disconnect between political decisions and frontline realities, further fueling dissatisfaction. Third, irregular working schedules, particularly night shifts, were seen as barriers to work-life balance and physical health, while also negatively impacting social and family responsibilities. Frustration over the lack of professional influence in shaping healthcare policies was a recurring concern.

**Conclusions:**

This study identifies key challenges influencing HCP retention, emphasizing the importance of restoring trust through transparent communication and professional engagement in policy making. Rather than relying on overly generalized approaches, retention efforts should be tailored to clusters of professionals with shared working conditions. Additionally, this study highlights three key insights: the growing distrust in the healthcare system and in policy makers as significant aspect in HCPs’ experience, shifting generational attitudes toward work commitment, and the need for collaborative programs between professional schools and employers to enhance work preparedness. These findings underscore the need for systemic changes to support workforce sustainability.

**Supplementary Information:**

The online version contains supplementary material available at 10.1186/s12913-025-12659-z.

## Background

Healthcare systems worldwide are facing a major shortage of healthcare professionals (HCPs) across all disciplines, posing a significant threat to care quality [1–4]. In many countries, healthcare institutions need to find solutions to maintain adequate staffing levels while contending with high turnover rates, an increasing number of HCPs leaving their profession and, in some cases, such as Switzerland, a limited capacity to train new professionals, which further exacerbates workforce shortages [5, 6].

The shortage of HCPs can be attributed to multiple interconnected factors including the growing demands of an aging population, increasing workloads, and financial constraints that negatively impact working conditions. These challenges contribute to a tense working climate and a loss of meaning at work ultimately leading to disengagement from HCPs [7–9]. The COVID-19 pandemic has further intensified these issues, placing additional stress on HCPs, and increasing burnout [10].

Factors contributing to HCPs’ intent to stay in their profession have been extensively studied. The most frequently cited factors include job satisfaction, work-life balance, workload, professional development opportunities and burnout [11]. However, translating these findings into concrete actions remains challenging for two main reasons. First, most research has focused primarily on nurses and physicians, making it unclear whether broad, interprofessional interventions would be effective across all healthcare professions [12, 13]. For example, studies comparing different professional groups have shown that while many of the concerns raised by professionals are similar, their impact on intent to stay varies by profession [13, 14]. A holistic interprofessional approach is essential for developing comprehensive actions that address workforce challenges across the healthcare system. In Switzerland, the SCOHPICA-HCP cohort, which includes HCPs from over 30 professions and investigates career trajectories and HCPs intention to stay in their profession, highlights the importance of such an approach [15]. Second, designing effective actions requires in-depth insight into HCPs’ experiences of working conditions in a shortage context and their association with job satisfaction, well-being, and motivation to remain in the profession. However, most qualitative studies addressing this issue have relied on in-depth interviews with small sample sizes, limiting comparative analysis across professional groups [16]. With advances in computational text analysis, it is now possible to analyze textual data from larger samples, particularly form open-ended survey responses [17–19]. Open-ended comments provide contextualized feedbacks and suggestions, allowing participants to express concerns in their own words while capturing insights from large and diverse professional groups. Furthermore, these open-ended responses are often more concrete and actionable for stakeholders, making them a valuable tool for informing workforce policies and retention programs [17, 20].

The aim of this study is to provide a comprehensive, interprofessional understanding of salient factors emerging from HCPs’ work experiences, identifying challenges that should be addressed to develop actions aimed at enhancing their intent to stay in the profession. To achieve this, we analyze open-ended comments from a self-reported survey within a large Swiss cohort study focused on HCPs intention to stay in the profession.

## Methods

This study draws on data from SCOHPICA-HCP, a national prospective open cohort examining the factors and career trajectories that influence Swiss HCPs’ intention to stay in their profession [15]. Specifically, the current study analyses data from the open-ended comment section at the end of the SCOHPICA-HCP survey, where participants were invited to provide comments about any aspects of their professional situation that they considered important and were not fully addressed in the survey.

### Population and data collection

The SCOHPICA-HCP cohort collects data from different types of HCPs (e.g., physicians, nurses, nurses’ aides, paramedics, physiotherapists, dieticians, pharmacists, etc.) practicing in Switzerland and working in direct contact with patients across different healthcare settings (e.g., hospitals, private practices, clinics, nursing homes, community services, etc.), irrespective of their employment status. HCPs were not directly contacted through their employer or workplaces; instead, recruitment occurred primarily via professional associations, state health institutions and the SCOHPICA website. Participants were invited to complete a self-administrated survey, which was directly accessible through the cohort’s website. The survey, described in detail elsewhere [15, 21], was not specifically developed for the present study and included approximately 140 questions. It concluded with an open-ended comment section where participants could share additional insights about their professional situations that they considered of importance and were not covered by the survey. This article focuses exclusively on the analysis of textual data from participants who provided open-ended comments.

All participants gave informed consent prior to completing the survey. After providing consent, participants were free to skip questions or stop the survey at any point. The SCOHPICA-HCP project was approved by the Cantonal Research Ethics Committee Vaud (CER-VD), Switzerland (project ID: 2022-01410), and is registered with ClinicalTrials.gov (identifier NCT05571488; first registered on 2022-10-07; updated on 2023-12-22).

### Material and analyses

Analyses concerned textual data from the open-ended comment section of the baseline survey conducted in the fall of 2022 and 2023. First, comments were anonymized by removing explicit names of individuals, institutions, or any identifying information. To ensure consistency across languages, responses in German, Italian, or French were translated into English by a professional translation company.

Computer-assisted textual analyses were carried out using IRaMuTeQ (version 0.7 alpha2, 2008–2014 Pierre Ratinaud), a Computer-Aided Qualitative Data Analysis Software specifically designed for analysing large textual datasets. The software offers a range of analytical tools based on classical lexical analysis methodologies. In this study, we applied the Reinert method, which allows for the extraction of thematic classes from large textual corpora. This method has been widely used across various research domains, including studies on patients and professionals’ experience, as well as public health research [22–24]. According to this method, texts are divided into elementary contextual units (ECUs)—typically sentences or short phrases—serving as the basis for analysis. A hierarchical descending classification, based on words co-occurrences, is then performed, grouping work into thematic classes. This process generates a dendrogram (tree graph) illustrating the relationships between thematic classes. Each class is associated with a distinct vocabulary and with representative textual excerpts, facilitating theme identification. To ensure robust text processing, the software utilizes pre-existing lexical dictionaries (integrated within IRaMuTeQ) to lemmatize words (grouping words based on their root form) and to identify accepted expressions (identifying multi-word phrases commonly used in healthcare contexts). Additionally, manual refinements are conducted to accurately classify profession-specific and domain-specific vocabulary.

Once the classification was completed, two researchers specialized in qualitative analysis independently examined the results, including representative words and excerpts, and assigned labels to thematic classes. To ensure accuracy, they revisited the original comments to contextualize the identified themes. Any discrepancies in interpretation were resolved through consensus discussions.

The software also enables the inclusion of latent variables to determine whether specific (sub)themes are over- or under-represented in particular participant groups (e.g., professional groups, work contexts) using a chi-square test with one degree of freedom. For this analysis, we included several variables from the SCOHPICA-HCP survey, including data collection year, linguistic region, professional group, work setting, gender, age group, presence of dependent children, informal caregiving status, years in profession, work schedule type, managerial role, employment rate, intent to stay in the profession, job satisfaction, number of employer changes, perceived work meaning, self-reported health, and burnout symptoms. A statistically significant over- or under-representation of a variable in a thematic class (determined by the chi-square test) indicates that the theme is mentioned more or less frequently by that group. However, a non-significant result does not imply the theme is absent in that group’s responses; rather, it suggests that its frequency of mention does not differ significantly from other groups.

## Results

### Sample and participant characteristics

Among the 5,927 participants who participated in the cohort for the first time in 2022 (*n*=1707) and 2023 (*n*=4220), a total of 1,811 participants provided an open-ended comment that was included in this study (612 in 2022 and 1,199 in 2023). These 1,811 comments comprised a total of 88,495 words for analysis.

Compared to the full SCOHPICA cohort sample, participants who left a comment exhibited slight but statistically significant differences in certain characteristics. Specifically, those who provided comments were slightly older and had greater professional seniority. They were also more likely to work in institutions, hospitals, or across multiple healthcare settings. Additionally, nurses and allied health professionals were overrepresented among those who left comments. Furthermore, this group reported lower intent to stay in the profession, lower job satisfaction, and more symptoms of burnout than those who did not provide comments. The characteristics of both groups, along with chi-square test results, are presented in Table 1.

**Table 1:** Characteristics of respondents’ who left a comment compared to the whole sample

Characteristics	First-time respondents in 2022 and 2023 (%)	First-time respondents in 2022 and 2023 who wrote a comment (%)	Chi2 difference
N	5,927	1,811	--
Gender			3.68 ns
Women	77.8	79.8	
Men	20.9	19.2	
Other/Prefer not to answer	1.3	1.0	
Age			9.78^**^
35 or less	35.0	32.8	
36 to 50	39.1	37.5	
More than 50	25.9	29.7	
Linguistic region			1.69 ns
French	52.9	52.6	
Italian	5.0	4.3	
German	42.1	43.1	
Work context			32.6^***^
Practice	14.5	12.1	
Home care	9.2	8.0	
Hospital	41.2	43.0	
Institution	7.7	10.3	
Pharmacy/lab	3.4	3.0	
School	1.2	2.1	
Emergency	5.8	4.9	
Multiple	14.6	15.1	
Other	2.4	1.5	
Profession			24.6^***^
Nurse	52.7	55.9	
Physician	12.2	12.5	
Allied health professions	15.9	17.7	
Medical-technical	2.1	1.8	
Pharmacist	2.6	2.6	
Assistant	4.6	2.9	
Psychologist/psychotherapist	1.9	2.4	
Paramedics	6.1	4.2	
Other	1.9	--	
Work rate			134.3^***^
Full	34.3	49.7	
Partial	65.7	50.3	
Informal caregiver role			119.9^***^
Yes	34.96	21.2	
No	65.04	78.8	
Longevity in the profession			7.3^*^
5 years and less	24.1	22.1	
6 to 15 years	35.3	33.8	
16 and more	40.6	44.1	
Type of schedules			3.9 ns
Days only	60.6	62.2	
Nights only	1.10	1.5	
Days and night	38.3	36.3	
Manager role			1.1 ns
Yes	27.9	26.6	
No	72.1	73.4	
Intent to stay in the profession			18.34^***^
Yes	68.4	63.0	
No	31.6	37.0	
Job satisfaction			24.5^***^
Hight	72.9	66.9	
Low	27.1	33.1	
Number of employer changes			18.6^***^
0 or 1	33.9	29.5	
2 or 3	32.2	31.5	
4 and more	33.9	39.0	
Meaning of work			0.6 ns
Hight	58.3	59.3	
Low	41.7	40.7	
Self-reported health			3.5 ns
Good	39.9	37.9	
Moderate	43.9	44.3	
Bad	16.2	17.8	
Burnout symptoms			10.2^***^
Yes	37.3	41.5	
No	62.7	58.5	

Note : ^*^p < .05; ^**^p < .01; ^***^p < .001

### Thematic analysis

The thematic analysis identified three main themes: (i) education and career path, (ii) working conditions, and (iii) working schedules. The firsts two were further divided into eight subthemes. The depth of elaboration on each theme and subtheme varied depending on the nature of participant responses. In some cases, participants provided only brief, descriptive statements without further explanation. As a result, some themes and subthemes are more developed than others in the following sections, as they are presented exactly as expressed by participants, without additional interpretation, to maintain the integrity of the qualitative analysis.

**Fig. 1:**
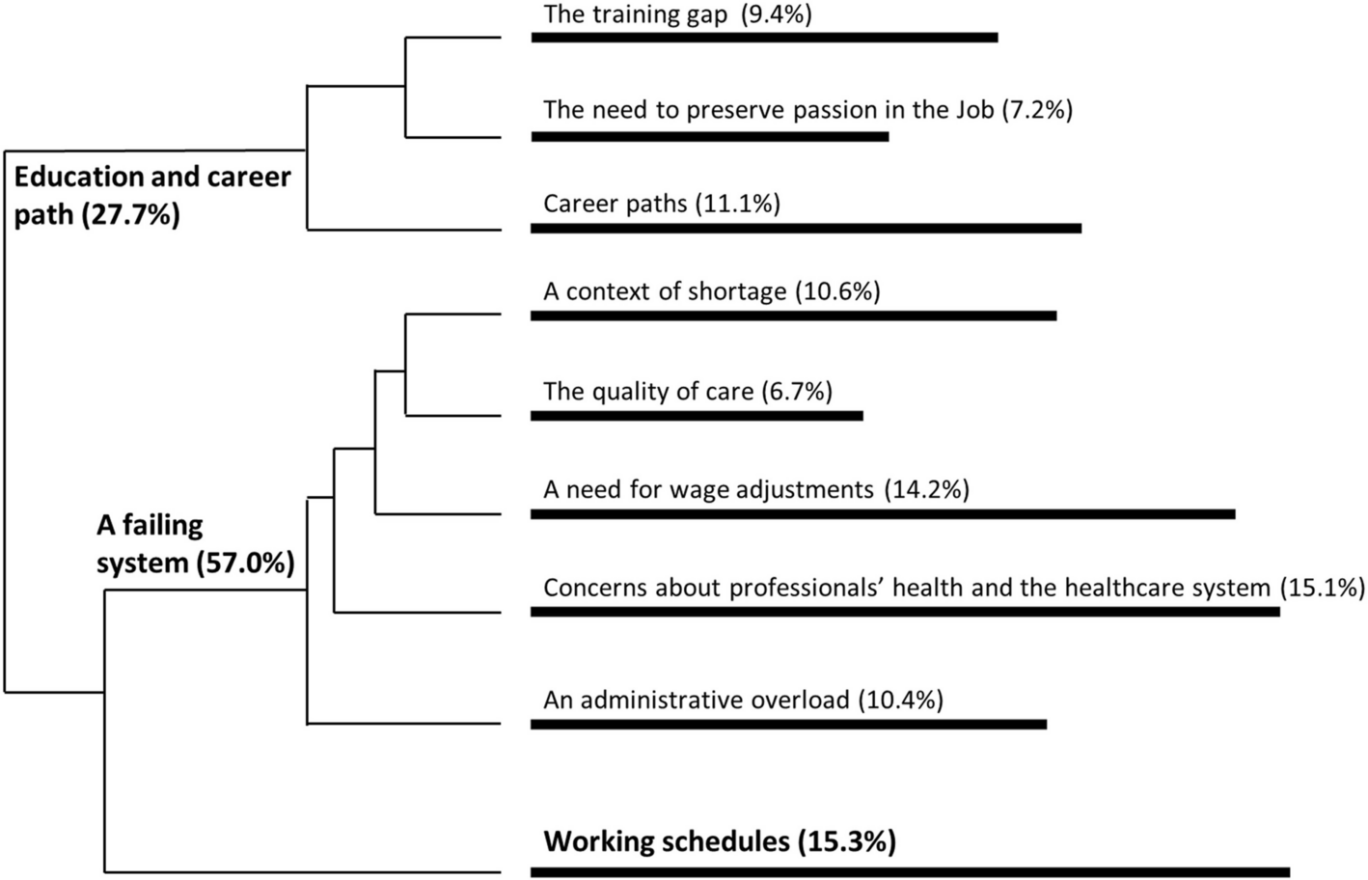
Dendrogram of main themes and subthemes extracted from open-ended comments

Figure 1 illustrates the relationship between main themes and subthemes, while Table 2 provides an overview of typical words and excerpts associated with each theme and subtheme. The chi2 values associated with the over-representation of certain respondent groups in themes and subthemes are presented in Additional File 1.

**Table 2:** Typical words and excerpts from main themes, subthemes

**Theme**	**Sub-theme**	**Typical words**	**Typical extract**
Education and career path	The training gap	*Train, master, degree, course, science, university, bachelor, diploma, apply*	*“I find the job beautiful and enjoy it. However, university of applied sciences training should prepare better for everyday professional life.”*
	The need to preserve passion in the Job	*Happy, passion, job, enjoy, suit, field, retire, great, team, supervisor*	*“The passion, I am very enthusiastic about my work and I love the work in itself, however the current conditions make it difficult to enjoy my job.”*
	Career paths	*Year, month, ago, change, job, retire, answer, last, end, leave, retirement, resign*	*“I have changed jobs since January 2023 because working conditions and values no longer met my expectations. If done 1 year ago, this same questionnaire would have had very different answers.”*
A failing system	A context of shortage	*Staff, long, treat, know, colleague, healthcare, term, system, disease, person*	*“The healthcare system is very disrupted. Everywhere there is a lack of staff and the number of patients and their high expectations/demands are constantly increasing.”*
	The quality of care	*Care, quality, money, safety, resident, resource, wrong, ensure, human, achieve*	*“healthcare workers are asked to do more (care, etc.) but without having the necessary resources, especially human, to achieve this, and this results in less and less quality care.”*
	A need for wage adjustments	*Rate, wage, salary, low, cost, increase, pay, live, value, responsibility, adjustment*	*“Wages are too low compared to the responsibilities, there is a lack of staff”*
	Concerns about professionals’ health and the healthcare system	*Health, professional, general, impact, insurance, find, exhaustion, important*	*“We become health professionals for a part of humanity but it is not recognised by decision makers resulting in a loss of meaning.”*
	An administrative overload	*Administrative, report, admin, spend, insurance, treatment, task, bill, patient*	*“The increasingly significant part of administrative work (including reporting to insurance companies) and the time it takes decreases the time I can spend with patients, decreases my energy and greatly alters my pleasure in practicing my profession.”*
Working schedules		*Day, hours, night, shift, schedule, holiday, week, weekend, work, overtime*	*“we work based on irregular schedules 365 days/365, day, night, weekends, holidays. With this working time, it's not possible to be part of society”*

The following sections detail the content of each thematic class according to this structure.

### 1. Education and career path

In this first main theme, participants described their training and career trajectories, often highlighting the difficulties they encountered. This theme was divided into three subthemes: 1) the training gap, 2) the need to preserve passion in the job, and 3) career interruptions.

#### The training gap

This subtheme, representing 9.4% of classified text units, highlighted three main gaps in the education and training system. The first gap concerned pre-graduate education, which participants criticized for insufficiently preparing students for the realities of the profession *(“university of applied sciences education should prepare better for everyday professional life”*). Many felt that essential aspects of their work, referred to as “*basic”* or “*necessary knowledge”*, were often overlooked. This created a disconnect between education and job demands, which some believed contributed to early departures from the profession (*“Training institutions neglect basic knowledge, whereas the 'unnecessary' is taught in the BSc […]. Large discrepancy between training and professional activity (resulting in professional withdrawal or burnout)”*).

The second gap related to post-graduate education, where financial and time constraints were identified as significant barriers to continuing professional development (*“In Switzerland, it's still very difficult to attend further training without financial support or to be able to continue training”*). Despite the importance of postgraduate training for skill enhancement and career development, participants reported limited opportunities for professional growth (*“I left the profession of paramedic due to little career opportunities and accessibility to postgraduate courses”*). Furthermore, some participants felt that their newly acquired skills were undervalued, as postgraduate training did not lead to salary increases, professional recognition, or opportunities to apply enhanced expertise: (*“In the healthcare sector there are no possibilities for keeping your salary while undergoing professional/technical further training, as only one function is taken into account in the performance-based wages. Specialist knowledge and expertise are not taken into account”).*

The third gap involved diploma recognition. Participants reported difficulties in obtaining recognition for diplomas earned abroad or in other Swiss cantons (*“I hold a master’s degree in public health from Belgium. This master's degree is not recognised in my institution, yet I was recruited precisely because I had this diploma”)*, which in some cases affected salaries and the possibility to use the full scope of practice.

HCPs who plan to stay in the profession, report job satisfaction, work across multiple healthcare settings, are based in the German-speaking part of Switzerland and have between six and 15 years of professional experience, are overrepresented in this subtheme.

#### The need to preserve passion in the job

This subtheme, representing 7.2% of classified text units, focused on the perception of healthcare as either a “vocation” (“*Overall, the nursing profession remains for me a vocation and currently, I don't see myself working in any other field*”) or a “passion” (“*I am still passionate about this profession*”). However, these terms were not explicitly defined by participants and may carry different interpretations depending on the individual. Participants frequently used highly positive words such as “*passion*,” “*happy*,” “*joy*,” “*love*,” “*great*,” and “*flourish*” When describing their professional experience. However, these expressions often appeared contrast with negative aspects of their work environment (“*I am very enthusiastic about my work, and I love the work in itself. However, the current conditions make it difficult to enjoy my job*.”). Some participants noted that the only way to “*practice with passion*” was to either become self-employed or switch to a different professional setting (“*I left my position in the public sector because the working conditions were appalling and I might even have left the profession if I had not found this new job*”; “*I am finally happy because I left my job to become self-employed as a complementary therapist recognised by the canton which is what I am passionate for*”). In addition to these challenges, participants observed a shift in motivations among HCPs. Many felt that fewer colleagues, especially younger ones, approached the profession with the same level of passion. For some participants, this difference in professional posture among the younger generation is associated with a lower quality of care. *(“In my opinion, generational conflicts are increasing, and the gap is bigger and bigger, which complicates relationships between colleagues and the care provided to patients, because some young people […] they don't have the passion for the job”*). Participants working in the ambulatory sector, satisfied with their current job, expressing a high work meaning, less than 35 years old and having worked for less than five years, are overrepresented in this subtheme.

#### Career paths

This subtheme, representing 11.1% of classified text units, focused on career breaks and changes of employers, most of which had occurred recently. While some comments simply described past or current job changes, others provided explanations for these changes. A frequent reason cited was (early) retirement (*“I have been retired since summer, now I only work occasionally”*). Burnout was also commonly mentioned as a cause for leaving a position (*“I had to leave my previous job […] because of total burnout. I have repeatedly considered leaving the profession due to exhaustion”*). Additionally, dissatisfaction with working conditions was another contributing factor (*“I left the hospital environment more than 10 years ago because I was already fed up with the deterioration of our working conditions”*). Some participants also cited childcare responsibilities as a reason in career interruptions. Beyond describing their professional changes, some participants expressed regret over the transformation of their profession over the years (*“I have experienced a massive change in healthcare and in my professional duties over the last 35 years”*). Participants over 50 years old and those who had worked in their profession for more than 16 years were overrepresented in this subtheme.

### 2. A failing system

The second major theme addressed the perceived deterioration or systemic failures within the healthcare system. It was divided into five subthemes: 1) staff shortage, 2) quality of care, 3) wage recognition, 4) concerns about professionals’ health and the healthcare system, and 5) administrative overload.

#### A context of shortage

This subtheme, representing 10.6% of the classified text units, highlighted the significant shortage of HCPs, which participants felt was exacerbated by an aging population with increasing complex care needs and higher patient expectations (*“Everywhere there is a lack of staff and the number of patients, and their high expectations/demands are constantly increasing”*). The shortage created a stressful work environment, characterized by high pressure, and, in some cases, mistreatment from employers (*“However, the pressure is high, the demand is high, and the job is emotionally demanding”; “I had to quit because I couldn't cope with the way patients and staff were treated”*). A key consequence of the shortage was a decline in care quality and an increased risk of errors (“*staff shortage in the service is not only very exhausting, but there are also increased errors which can lead to dangerous care*”). Many participants also expressed concern for patient safety, particularly due to the increasing reliance on low-skilled professionals to fill staffing absences (“*poorly qualified staff is also a burden for long standing well qualified staff who leave the hospital setting out of frustration*”). Although some participants saw supportive colleagues as a valuable resource, they noted that this support was fragile, given the increase of absences in teams due to fatigue or burnout.

Participants perceived the shortage of HCPs as a systemic issue resulting from the current management of the healthcare system. Financial constraints, perceived as ill-considered, a lack of field knowledge and of communication were frequently mentioned as contributing factors (*“The healthcare system is very disrupted. […] Savings are made in the wrong places and the population is not properly informed - the policy is totally failing”)*. Many participants felt trapped in a system that had reached its limits and was resistant to change (“*However, due to lack of resources (time and staff) the quality of these side duties deteriorates because they are not recognised by the governing bodies who only seek to save money.*”). Participants reporting burnout symptoms, those dissatisfied with their job, and those not intending to stay in the profession, nurses, as well as participants who worked in institution (e.g. hospitals, clinics, nursing homes were overrepresented in this subtheme.

#### The quality of care

This subtheme, representing 6.7% of the classified text units, was closely linked to the issue of staff shortages. Participants described how poor working conditions negatively affected both care quality and patient safety. They noted that insufficient financial and human resources limited their ability to provide proper care, which in turn reduced their motivation and capacity to perform effectively (*“the working environment deteriorates, motivation decreases, and a let go dynamic sets in, which ultimately undermines the quality of care and patient safety.”).* This subtheme was no further elaborated. Participants with low job satisfaction were overrepresented in this subtheme.

#### A need for wage adjustments

This subtheme, representing 14.2% of the classified text units, focused on concerns about salary recognition. Participants felt that salaries had not kept pace with inflation, leading to financial strain, particularly for those with dependent children. Salary negotiations were described as challenging or impossible, contributing to dissatisfaction and a sense of being undervalued (*“The salary I receive absolutely does not correspond to my level of responsibility. It undervalues me in my practice, gives me a feeling of powerlessness and uselessness within the hospital. If this does not change quickly, I am thinking of changing professions in the medium term, because the increase in the cost of living no longer allows me to be confident in my daily budget.”)*. Participants also criticized salary discrepancies among colleagues with the same qualifications and experience but working in different contexts or cantons. (“*Wages vary greatly in the healthcare sector, depending on the Canton and on the healthcare institution. Why not having the same? Same education and same experience/further training should also be paid accordingly, in the same way!*”).

Many attributed low salaries and inequities to an outdated ambulatory billing system, which they felt failed to reflect evolving healthcare practices, professional demands, or the cost of living (*“Physiotherapists earn very little already, our tax points should at least adjusted to inflation, which has not happened for 25 years”*; *“Significant income loss as a result of the continuous decrease in the value of the Tarmed point scale”*). Participants expressed frustration with policymakers, believing they had failed to anticipate or address these salary-related challenges, which contributed to demotivation and job dissatisfaction. Participants working in the ambulatory sector or emergency wards, those reporting low work meaning, and those experiencing burnout symptoms were overrepresented in this subtheme.

#### Concerns about professionals’ health and the healthcare system

This subtheme, representing 15.1% of the classified text units, focused on two interconnected concerns: the health risks faced by professionals and the sustainability of the healthcare system. At the individual level, participants described high mental health risks associated with their profession and regretted that these risks were not addressed during training (“*To what extent in our training we are made aware of the risk of professional burnout and how we protect health*.”). The psychosocial impact of the job was emphasized, with participants describing an accumulation of stressors that undermined job satisfaction. These stressors included stress, exhaustion, work overload, poor working conditions, and lack of recognition (*“My exhaustion is due to an accumulation of things, including stress at work”*; *“stress, exhaustion, desire to leave the profession or workplace is due, in my case, to societal requirements and policies that prevent me from providing quality care while developing all my skills”*). At the systemic level, participants expressed deep concern that the healthcare system was reaching a breaking point. that political and financial agendas ignored realities in the field, restricting resources needed for quality care. This disconnect led to frustration, discouragement, and distrust toward decision-makers *(“we become health professionals for a part of humanity, but it is not recognised by decision makers resulting in a loss of meaning”*). These frustrations weighted on both participants’ motivation to stay in the profession and personal health (“*I feel that the system is collapsing and despite my commitment to professional policy, I feel powerless, and this especially impacts on my health*.”). Nurses, physicians and medicotechnical professions were overrepresented in this subtheme.

#### An administrative overload

This subtheme, representing 10.4% of the classified text units, highlighted the burden of administrative tasks which participants viewed as time-consuming, inefficient, and unrelated to patient care. Examples included medical reports, electronic patient records, treatment service invoices, and documents required by insurance companies. These tasks were seen as outside their professional responsibilities and a drain on time that could be spent on actual care (“*an unnecessary increase in admin and paperwork, which consumes time that could be used…for the actual therapy”).* They described the increasing complexity of digital tools and paperwork requirements, which reduced their ability to focus on direct patient interactions. (*“The greatest dissatisfaction for me is the time-consuming and senseless increase in digital tools, administrative rules and guidelines for documentation and billing and time to use and respect them.”*). Two main factors were identified as contributing to this issue: i) the lack of administrative staff to handle operational tasks and ii) the increasing demands for justification from health insurance companies, which participants felt undermined their autonomy (“*The many rapid and unnecessary changes are characterised by fear and control; this is certainly overregulation, and ultimately it's a burden for both practitioners as well as patients.”)* The consequences of these administrative demands included less time for patient care and fewer opportunities for direct interaction with patients (*“The increasingly significant part of administrative work (including reporting to insurance companies) and the time it takes decreases the time I can spend with patients”*). Participants also criticized the bureaucratic structure of the healthcare system, which prioritized profitability over the fundamental purpose of care. They felt that the pressure for efficiency conflicted with their vision of healthcare and added to their frustration (*“what's painful and exhausts me are the institutional one-size-fits-all approach which loses the purpose of care, the billing, we must be profitable, at least 5 hours worked per day in addition to the rest”*). Participants in practice-based settings, particularly physicians, physiotherapists, or psychologists, as well as those with more than 15 years of experience were overrepresented in this subtheme.

### 3. Working schedules

This theme, representing 15.3% of the classified text units, addressed the issue of working hours and scheduling. Irregular hours, particularly night shifts, were identified as a major concern, significantly impacting quality of life. Participants reported adverse effects on physical health, including disrupted sleep patterns and severe fatigue (*“The difficulty of schedules and the lack of recovery time (additional days of recovery leave in case of evening or night work as in the educational environment)”*). Social life was also heavily affected, leaving participants feeling disconnected from society and experiencing strained relationships (*“Because we work based on irregular schedules 365 days/365, day, night, weekends, holidays. With this working time, it's not possible to be part of society”*; *“Especially after specific working days, I am tired and exhausted, and I don't have any energy left to maintain any leisure activities or private social contact outside the family”*). Family responsibilities and childcare management posed additional challenges (*“Support for working mothers is always scarce. Currently, I can't rely on any nursery providing flexible hours for people working shifts”*). Participants with caregiving responsibilities at home also described organizational struggles caused by their demanding work schedules. Many felt that these difficult working conditions, especially irregular hours, warranted better financial compensation, which they believed was currently inadequate. Participants working as nurses, those working in hospitals or in home care, women and participants with children were overrepresented in this subtheme.

## Discussion

This study provides key insights into the challenges faced by HCPs in a shortage context across various roles and settings. Three main themes emerged: education and career path, the perceived failure of the healthcare system, and issues related to working schedules. These findings offer an overview of the multifaceted work-related concerns currently raised by HCPs in Switzerland when reflecting on their willingness to stay in their profession. They highlight the complexity of the situation, involving multiple levels of action over which HCPs have limited control. These challenges are not unique to Switzerland; similar issues have been reported in other high-income countries, such as Australia, European countries, or Canada [25–29]. However, Switzerland faces additional constraints, including limited training capacities and a strong reliance on foreign-trained HCPs, further complicating workforce sustainability.

Regarding education, participants raised concerns that pre-graduate training does not sufficiently prepare students for the realities of professional practice. This issue is well-documented in the literature. For instance, in Switzerland, a study found that 54% of medical residents felt their curriculum had not adequately prepared them for clinical practice [30]. Similar findings have been observed internationally across various healthcare professions [31–33], particularly during the first year of practice [34, 35]. Some studies even suggest that the overall level of preparedness among new professionals has declined in recent years [36, 37]. This issue is critical as it contributes to early departures from the profession [38]. Efforts to address this problem have focused on improving both the actual preparedness and new graduates’ perception of being prepared. Key recommendations include raising awareness among institutions about the challenges of transitioning from education to practice and training managers to adopt a coaching role for new graduates [39, 40]. In nursing, transition programs combining teaching sessions and mentoring during the first year of practice have shown promising results in supporting newly graduated HCPs [41, 42]. These programs not only strengthen young professionals’ sense of belonging within their teams [43] but also positively impact on turnover rates and retention [44]. Despite their benefits, few institutions invest in such programs due to financial and human resource constraints [45, 46]. Partnering with educational institutions to develop and implement these programs in workplaces could be a practical and effective solution [47].

Another key finding in our results is the critical view and distrust HCPs have toward the healthcare system. Trust in management has long been recognized a major determinant of HCPs retention [48], particularly during crises like the COVID-19 pandemic [49, 50]. However, as in our, recent studies highlight a growing distrust not only toward direct management but also toward institutions and the broader healthcare system, questioning its ability to address major public health challenges [51]. The perception of a system that has become “too big to care” [52]-failing to meet both care-related and workplace needs - significantly contributes to this trust crisis. To our knowledge, no recent study specifically examined this question among Swiss HCPs, making it difficult to determine whether our findings reflect a localized or global concern. Nonetheless, restoring trust between HCPs and decision-makers is now seen as a priority [50]. One approach is to improve transparent communication between patients, HCPs, and healthcare decision-makers [53]. Another involves increasing professional participation in policymaking, particularly in working conditions and broader care models, using co-production approaches [54]. Advocates of co-production argue that focusing solely on working conditions or well-being at work is insufficient to fully understand the shortage crisis. To them, HCPs operate within a broader social and political context, which has evolved over time [54]. The current crisis stems from outdated norms and regulations that no longer align with this new social reality. This misalignment has contributed to a loss of meaning and a diminished sense of professional agency [54], concerns that were echoed by SCOHPICA participants. Several themes from our findings illustrate this disconnect, including criticism of remuneration systems. Participants noted that while their responsibilities and skills have increased with advances in education and practice, their salaries have not kept pace. In the literature, salary has long been a source of dissatisfaction, especially for nurses. However, before 2010, its influence on intentions to stay or leave the profession was debated [55]. More recent studies, particularly among nurses and physicians, highlight salary as an increasing important factor to consider [11].

Another change observed in our findings concerns the refusal of excessive work commitment in favour of a better work-life balance. This shift is accompanied by a preference for flexible working schedules, improved recognition, and engagement in meaningful tasks. It challenges the long-standing perception of healthcare as a vocational calling [56], where professionals are expected to "live to work." Instead, an increasing number of HCPs are adopting a "work to live" mindset, emphasizing the importance of work-life balance, self-care, and sustainable work practices [57]. This new mindset raises critical concerns about the long-standing over-reliance of healthcare institutions on individual overcommitment [57]. In many care settings, staff shortages or unexpected absences have historically been managed by requesting HCPs to reschedule shifts or return from vacation, thereby relying on their willingness to overinvest in work. In our findings, many participants expressed a growing reluctance to take on additional tasks beyond their core responsibilities citing a perceived lack of institutional support or willingness to contain excessive workloads by means other than team involvement. This emerging pattern aligns with "quiet quitting," a phenomenon recently described in workplace studies following the COVID-19 pandemic [58]. Quiet quitting does not refer to leaving one's job, but rather to withdrawing from non-mandatory tasks while continuing to fulfill core job responsibilities [57]. In healthcare, this behavior is particularly relevant given the high emotional and physical demands of the profession. For HCPs, quiet quitting often manifests as a reduction in voluntary contributions, such as staying late for emergencies, mentoring junior staff, or assuming additional administrative duties beyond their contractual obligations [59]. Recent research suggests that quiet quitting is more prevalent in high-burden professions, particularly those experiencing widespread burnout, administrative overload, and a lack of institutional recognition [57, 59–61]. In our study, participants frequently expressed frustration over being expected to take on extra responsibilities without corresponding support, recognition, or compensation. These findings resonate with workplace studies indicating that quiet quitting is often a response to the perception of unfair labor expectations rather than simple disengagement [57, 60]. While quiet quitting is considered as disruptive to care organization, it can be seen as a symptom of deeper systemic failures [57]. This phenomenon underscores structural inefficiencies, revealing an overreliance on professionals’ overcommitment rather than sustainable workforce strategies [57]. It also raises concerns for future workforce sustainability, particularly as younger professionals are less willing to sacrifice personal well-being for professional demands [61, 62]. The shift in work commitment among younger HCPs, particularly their prioritization of work-life balance, has been observed globally [63, 64]. However, in Switzerland, this shift is exacerbated by a lack of flexible working arrangements [65] in contrast to countries such as Scandinavian countries, where part-time and adaptive schedules are more commonly integrated into healthcare systems [66, 67]. Our findings do not explicitly indicate that younger professionals are more prone to quiet quitting, but some participants perceived a generational shift in attitudes toward work commitment. In some comments, younger colleagues were described as less dedicated to the profession, what was perceived as a threat to its vocational culture. These intergenerational tensions reflect broader workplace trends where traditional notions of professional duty are being redefined. These situations may strain relationships in teams and between managers and teams potentially impacting collaboration, job satisfaction, and overall team cohesion. Given the critical role of collaboration in healthcare settings, further research is needed to explore how these shifts in work commitment impact team cohesion, patient care, and long-term workforce retention.

Our results also revealed differences among professional groups. For instance, concerns regarding staff shortages, professionals’ health, the healthcare system, and scheduling issues were particularly raised by nurses. However, findings suggest that differences in working context may play a more significant role than professional group differences in shaping certain attitudes. Indeed, professionals across different groups can share similar working realities, leading to comparable perspectives on job retention [13]. Our results suggest that while it is important to acknowledge differences between professional groups when addressing retention, it is not necessary to develop entirely separate strategies for each profession. Instead, a more effective approach could involve identifying clusters of professionals who share similar working conditions, such as context, salary, or career development patterns. Efforts to address healthcare workforce shortages should therefore prioritize tailored solutions for these clusters rather than relying on overly generalized policies or highly specific interventions targeting individual groups. his context-sensitive approach would better align with professionals’ actual needs and priorities, ultimately supporting workforce retention more effectively.

### Strengths and limitations

This study has several strengths. First, it provides a broad overview of insights from a diverse sample of HCPs working in various professional contexts. Second, it employs computer-assisted textual analysis, enabling a systematic and efficient examination of a large number of spontaneous comments. This approach adds empirical value and highlight the key themes relevant to HCP retention. However, some limitations must be considered when interpreting the results. The first limitation concerns the recruitment and the representation of professional groups. Due to the absence of comprehensive HCP registries in Switzerland, participants were recruited indirectly through professional associations. While our study includes over 30 healthcare professions, some are underrepresentation in the sample because they were harder to reach. At the opposite, nurses and physicians were overrepresented. This overrepresentation is not unexpected, as nurses and physicians constitute the largest groups of HCPs in Switzerland (i.e more than 200’000 nurses and 42’000 physicians). However, this imbalance may limit the generalizability of findings to less-represented professions (e.g., paramedics, dietitians, occupational therapists) whose profession-specific challenges may be underexplored. Additionally, recruitment through professional associations, health institutions, as well as the use of an online open-access survey may have favored participation among groups with stronger networks or better digital access, while others may have been underrepresented due to lack of awareness. To improve representation in future research, strategies such as targeted outreach, diverse recruitment channels, incentives, and stratified sampling could be considered. The second limitation relates to selection bias, as only currently employed healthcare professionals who had the time and energy to participate responded. As a result, individuals who were on long-term sick leave or had already left the profession are underrepresented. To address this issue, the SCOHPICA cohort actively encourages HCPs who have left the profession to remain in the study. Furthermore, a dedicated project will specifically explore the experiences of former healthcare professionals, ensuring a more comprehensive understanding of workforce attrition in future research. The third limitation concerns the fact that only one-third of participants provided an open comment. Results indicate that participant who provided an open comment reported lower intent to stay, lower job satisfaction, and more symptoms of burnout. While this must be considered when interpreting the findings, it is also particularly relevant, as retention initiatives should primarily target these professionals. Their experiences provide critical insights into the factors that influence retention and should inform the development of interventions. Finally, the fourth limitation pertains to a common critique of computer-assisted text analysis—namely, the risk of losing contextual meaning due to text fragmentation. To mitigate this concern, researchers regularly returned to the original texts and validated interpretations through discussions to ensure accuracy and reliability in the thematic analysis.

## Conclusion

The aim of our study was to explore key work experiences influencing HCPs’ intention to stay in or leave their profession. We also sought to identify main challenges that could inform retention strategies, such as bridging educational and professional gaps, strengthening HCPs’ trust in the healthcare system, and allowing them to preserve their social life. We’ve also learned from our results that addressing healthcare professional shortages requires a multilevel approach with concrete recommendations at each level. At the institutional level, onboarding and mentoring programs could be systematically implemented in collaboration with professional schools and healthcare institutions. In the short term, institutions could enhance work flexibility by introducing self-scheduling for better shift management and facilitating internal mobility to support skills development and career progression. To help management navigate intergenerational challenges, institutions could strengthen managerial support through leadership coaching and sparring partners, equipping managers with the necessary tools to foster a more supportive and adaptive work environment. Additionally, work organization should be restructured, including the creation of new roles to prevent task redundancy and the loss of professional meaning. At the policy level, steps must be taken to bridge the disconnect between policymakers and frontline professionals. One approach could be the establishment of professional councils that actively involve healthcare workers in the development of healthcare policies, ensuring that reforms align field realities. Moreover, developing and funding national health workforce strategic and investment plans—as recommended by the World Health Organization’s guidelines on health workforce development—would provide a structured, long-term solution to workforce sustainability.

Our findings highlight pragmatic implications for healthcare workforce management and policy, emphasizing the need for multilevel interventions. By implementing evidence-based workforce strategies that align with both professionals’ expectations and system needs, healthcare institutions and policymakers can enhance retention, improve working conditions, and ensure high-quality patient care. Future research could build on our insights and recommendations to develop and assess the effectiveness of concrete interventions. Additionally, exploring intergenerational dynamics is essential, as they can impact management leadership and team cohesion—both key factors in professionals' long-term commitment to their careers. Additionally, further investigation into intergenerational dynamics is needed, as these factors can weaken management leadership and team cohesion—both of which are crucial factors for HCPs retention.

## Supplementary Information

Supplementary Material 1.

## Data Availability

Due to the nature of data and to ethic comity requirements concerning respondents’ identification, restrictions apply to the availability of these data, and they are not available.

## Abbreviation

HCPs: Health care professionals
