# Supplementary material for: Work experiences of healthcare professionals in a shortage context: analysis of open-ended comments in a Swiss cohort (SCOHPICA)
Source: BMC Health Serv Res. 2025 Apr 9;25:520. doi: 10.1186/s12913-025-12659-z (PMC11984188; doi:10.1186/s12913-025-12659-z)
Supplement: Supplementary file 1 — Supplementary Material 1. [file 12913_2025_12659_MOESM1_ESM.docx]

Additional File 1. chi2 values for over-representation respondent groups in themes and subthemes (all df = 1).

| Themes | Subthemes | Respondent groups | Chi2 | N |
| --- | --- | --- | --- | --- |
| Education and career path | The training gap | High intent to stay | 13.3^***^ | 1808 |
|  |  | High job satisfaction | 29.4^***^ | 1803 |
|  |  | Activity in muliple healthcare settings | 6.3^*^ | 1568 |
|  |  | German speaking | 24.9^***^ | 1811 |
|  |  | 6 - 15 years of experience | 6.3^*^ | 1798 |
|  |  |  |  |  |
|  | The need to preserve passion in the Job | Ambulatory sector | 4.5^*^ | 1568 |
|  |  | High job satisfaction | 11.0^***^ | 1803 |
|  |  | High work meaning | 5.7^*^ | 1807 |
|  |  | < 35 years old | 5.5^*^ | 1716 |
|  |  | < 5 years of experience | 4.5^*^ | 1798 |
|  |  |  |  |  |
|  | Career paths | < 50 years old | 9.1^**^ | 1716 |
|  |  | > 15 years of experience | 8.9^**^ | 1798 |
|  |  |  |  |  |
| A failing system | A context of shortage | Burnout symptoms | 9.6^**^ | 1763 |
|  |  | Low job satisfaction | 8.8^**^ | 1803 |
|  |  | Low intent to stay | 5.4^*^ | 1808 |
|  |  | Nurses | 15.2^***^ | 1732 |
|  |  | Hospitals | 15.4^***^ | 1568 |
|  |  | Nursing homes | 7.2^**^ | 1568 |
|  |  |  |  |  |
|  | The quality of care | Low job satisfaction | 8.8^**^ | 1803 |
|  |  |  |  |  |
|  | A need for wage adjustments | Ambulatory sector | 3.9^*^ | 1568 |
|  |  | Emergency wards | 4.4^*^ | 1568 |
|  |  | Low work meaning | 6.3^*^ | 1807 |
|  |  | Burnout symptoms | 11.1^***^ | 1763 |
|  |  |  |  |  |
|  | Concerns about professionals’ health and the healthcare system | Nurses | 4.1^*^ | 1732 |
|  |  | Physicians | 11.7^***^ | 1732 |
|  |  | Medicotechnical professions | 6.1^*^ | 1732 |
|  |  |  |  |  |
|  | An administrative overload | Practice-based settings | 89.3^***^ | 1568 |
|  |  | Physicians | 38.2^***^ | 1732 |
|  |  | Psychologists | 11.2^***^ | 1732 |
|  |  | > 15 years of experience | 12.1^***^ | 1798 |
|  |  |  |  |  |
| Working schedules |  | Nurses | 47.4^***^ | 1732 |
|  |  | Hospitals | 6.6. ^**^ | 1568 |
|  |  | Home care | 7.1^**^ | 1568 |
|  |  | Women | 3.9^*^ | 1793 |
|  |  | With children | 4.2^*^ | 1561 |

Note : * p<.05; ** p<.01; *** p<.001
